# Galbut Virus Infection Minimally Influences *Drosophila melanogaster* Fitness Traits in a Strain and Sex-Dependent Manner

**DOI:** 10.3390/v15020539

**Published:** 2023-02-15

**Authors:** Shaun T. Cross, Ali L. Brehm, Tillie J. Dunham, Case P. Rodgers, Alexandra H. Keene, Grace I. Borlee, Mark D. Stenglein

**Affiliations:** 1 Department of Environmental, Agricultural, and Occupational Health, College of Public Health, University of Nebraska Medical Center, Omaha, NE 68198, USA; 2 Center for Vector-Borne and Infectious Diseases, Department of Microbiology, Immunology, and Pathology, College of Veterinary Medicine and Biomedical Sciences, Colorado State University, Fort Collins, CO 80523, USA

**Keywords:** galbut virus, *Drosophila melanogaster*, DGRP, arthropod, virus–host interactions, fitness, virome, partitivirus, persistent viruses

## Abstract

Galbut virus (family *Partitiviridae*) infects *Drosophila melanogaster* and can be transmitted vertically from infected mothers or infected fathers with near perfect efficiency. This form of super-Mendelian inheritance should drive infection to 100% prevalence, and indeed, galbut virus is ubiquitous in wild *D. melanogaster* populations. However, on average, only about 60% of individual flies are infected. One possible explanation for this is that a subset of flies are resistant to infection. Although galbut virus-infected flies appear healthy, infection may be sufficiently costly to drive selection for resistant hosts, thereby decreasing overall prevalence. To test this hypothesis, we quantified a variety of fitness-related traits in galbut virus-infected flies from two lines from the *Drosophila* Genetic Reference Panel (DGRP). Galbut virus-infected flies had no difference in average lifespan and total offspring production compared to their uninfected counterparts. Galbut virus-infected DGRP-517 flies pupated and eclosed faster than their uninfected counterparts. Some galbut virus-infected flies exhibited altered sensitivity to viral, bacterial, and fungal pathogens. The microbiome composition of flies was not measurably perturbed by galbut virus infection. Differences in phenotype attributable to galbut virus infection varied as a function of fly sex and DGRP strain, and differences attributable to infection status were dwarfed by larger differences attributable to strain and sex. Thus, galbut virus infection does produce measurable phenotypic changes, with changes being minor, offsetting, and possibly net-negative.

## 1. Introduction

Galbut virus is a remarkably successful persistent virus of *Drosophila melanogaster* [1,2,3,4]. Infected flies have been found on five continents, and every wild population that has been tested includes some infected individuals. This degree of success is attributable to efficient biparental vertical transmission: in some genetic backgrounds, infected mothers or infected fathers can transmit galbut virus to 100% of offspring, providing a means for infection to increase in frequency generation over generation [4,5].

Galbut virus is a partitivirus (*Partitiviridae*; segmented, double-stranded RNA genomes), a group of viruses generally known for mild, persistent infections [6,7,8,9]. Plant-infecting partitiviruses were originally called cryptic viruses (former genus *Cryptovirus*) because of their inapparent phenotypic effects [9,10]. Galbut virus infection is similarly cryptic: despite a century of *Drosophila* research and despite its ubiquity, galbut virus was only recently discovered by shotgun metagenomics [1]. Galbut virus-infected flies do not exhibit obvious phenotypic differences from their uninfected counterparts in the lab or in the wild [4].

Nevertheless, there are indications that galbut virus may be in conflict with its host. Although infection is ubiquitous, the fraction of infected flies ranges from 13–100% in different populations, and on average, only ~60% of flies are infected [1,3,4]. We also found that some *Drosophila* Genetic Reference Panel (DGRP) lines were relatively refractory to infection or vertical transmission over multiple generations [4,11]. Galbut virus sequences exhibited high ratios of non-synonymous-to-synonymous variation (high dN/dS ratios), which could be consistent with selection driven by host–virus conflict [1]. Verdadero virus, a partitivirus that infects *Aedes aegypti*, exhibited similarly efficient biparental vertical transmission in colonized mosquitoes but also is not at 100% prevalence in wild populations [12,13,14].

We hypothesized that galbut virus might exact a fitness cost that is small but sufficient to drive selection for resistant individuals. A fitness cost need only be greater than the reciprocal of a species’ population size for selection to act on it [15]. Thus, for an organism like *D. melanogaster* with a large population size, selection can act on very small fitness differences. This could limit the overall success of galbut virus and similarly “cryptic” persistent viruses in host populations. To test this hypothesis, we quantified a number of fitness-linked phenotypes in galbut virus-infected flies. Host genotype and sex are variables that can substantially influence the outcome of infection [16,17,18,19,20,21], so we evaluated the phenotype of galbut virus-infected males and females from DGRP strains 319 and 517.

Relatively little is known about arthropod-infecting partitiviruses, which have now been identified in association with a broad range of hosts [22,23,24,25,26,27,28]. Metagenomic surveys of apparently healthy, free-living organisms have in general produced a flood of new virus sequences [12,29,30,31]. However, beyond sequence description and phylogenetic placement, little is known about the biological impact of all of these newly recognized viruses. Follow-up virological studies that build upon this trove of sequence data are needed [4,28,32,33,34]. The ability to study a highly successful natural virus of a premier model organism represents a great opportunity to shed light on insect-infecting partitiviruses and persistent viral infections more generally.

## 2. Materials and Methods

*Drosophila* rearing and maintenance. Flies were reared on the Bloomington *Drosophila* Stock Center (BDSC) standard cornmeal diet (https://bdsc.indiana.edu/information/recipes/bloomfood.html, (accessed on 15 January 2021)). Stocks were housed at 25 °C and changed every 14 days. All experiments were performed with *Drosophila* Genetic Reference Panel (DGRP) stocks 399 and 517, acquired from the BDSC [11,35]. Generation of galbut virus-infected lineages derived from these lines by microinjection was described previously [4].

Experimental groups consisted of galbut virus-infected or uninfected DGRP 399 or DGRP 517 males or females (2 strains × 2 sexes × 2 galbut virus-infection status = 8 groups). All flies were 3–5-day-old virgins reared in a 12 h light/dark cycle at 25 °C unless otherwise stated. 

Quantification of galbut virus RNA levels. Total RNA was extracted from individual 5-day-old virgin flies using a bead-based protocol as previously described [4]. cDNA was synthesized by adding 5.5 µL of RNA to 200 pmol of a random 15 mer oligonucleotide and incubated for 5 min at 65 °C, then set on ice for 1 min. A reverse transcription (RT) mixture containing the following was added (10 μL reaction volume): 1x SuperScript III (SSIII) FS reaction buffer (Invitrogen, Waltham, MA, USA), 5 mM dithiothreitol (Invitrogen), 1 mM each deoxynucleotide triphosphates (dNTPs) (NEB), and 100 U SSIII reverse transcriptase enzyme (Invitrogen), then incubated at 42 °C for 30 min, 50 °C for 30 min, and at 70 °C for 15 min. Then, 90 μL of nuclease-free H_2_O was added to dilute the cDNA to a final volume of 100 μL. 

Following cDNA synthesis, qPCR reactions were set up using Luna qPCR Master Mix (NEB), following the manufacturer’s protocol. The qPCR reaction was performed on LightCycler 480 (Roche) under the following protocol: 95 °C for 3 min, 40 cycles of 95 °C for 10 s, then 60 °C for 45 s, followed by a melting curve analysis. Primer sequences can be found in Table S1.

Quantification of major microbiome constituent DNA levels. Total DNA was extracted from pools of ten 4–5-day-old virgin flies (3 pools per group). Flies were surface sterilized by vortexing in 70% ethanol for 2 min, followed by two rinses with autoclaved ddH_2_O and vortexing for 1 min. Flies were then stored at −80 °C until DNA was extracted. DNA was extracted using the DNeasy Tissue and Blood extraction kit (Qiagen, Hilden, Germany), following the manufacturer’s protocol for insect tissues with three modifications. First, samples were added to 180 μL ATL buffer (provided in kit) along with a single BB bead and homogenized using a Qiagen TissueLyzer for 3 min at 30 Hz rather than homogenizing by hand. Second, samples were incubated in proteinase K for a duration of 4 h. Last, following incubation with proteinase K, samples were treated with 20 μL of RNase A (2 mg/mL; Sigma Aldrich, St. Louis, MI, USA) for 30 min at 37 °C. After RNase treatment, samples were processed as stated in the manufacturer’s protocol. DNA was used as input for qPCR reactions that were performed as described above for galbut virus. Microbiome analysis primer sequences were predominately from Early et al. [36] and are listed in Table S1.

Lifespan and fecundity assays were performed essentially as previously described [37]. Flies were reared in five replicate groups of 10 adults (5 females and 5 males). Flies were checked daily for survival of adults, and living adults were moved to fresh media every 3 days. Longevity of adults was compared using the R survival package [38], which makes use of a Cox regression model. After adults were moved, original vials containing laid eggs were kept for 14 days, after which offspring were counted and sexed. The cumulative numbers of offspring among replicates were normally distributed (determined by Shapiro–Wilk test), so *t*-tests were used to compare the number of offspring produced by galbut virus-infected and uninfected flies.

Total egg production was measured by housing 10 male and 10 female flies in bottles with an apple agar plate coated with yeast paste (1:1 yeast and water) to promote egg laying (3 replicate bottles per group). Egg plates were replaced every 24 h, and the used plates containing eggs were frozen at −20 °C until the eggs were counted. Plates were collected each day for 3 days. This experiment was performed twice for a total of six biological replicates per group. Images of egg plates were captured, and eggs were counted manually using the ImageJ cell counter program [39]. Statistical significance between total eggs laid was performed using an analysis of variance (ANOVA) test. All fecundity measurements were analyzed with R scripts, which can be found at: https://github.com/scross92/galbut_fitness_analysis (accessed on 31 January 2023).

Developmental speed assays were performed as previously described [40]. Eggs were collected using standard apple agar plates without preservative, with a mixture of 1:1 yeast and water applied. Every hour for 6–8 h, agar plates were discarded and replaced to encourage egg synchronization. Agar plates were replaced a final time and incubated for several hours. The plates were removed, and eggs were collected using an autoclaved brush. Twenty eggs were collected and moved to non-nutritive agar plates containing 5% sucrose/2% agar with no antimicrobials added (no tegosept). An agar plate was placed inside a larger petri dish with a damp paper towel on the bottom and moved to a 25 °C incubator with a 12 h light/dark cycle. Every 2 days, yeast paste was added as a nutrition source for developing flies. Yeast were killed prior to use in the paste by microwaving for 45 s on high to prevent overgrowth. Plates were checked daily for pupae to determine speed of pupation. Once pupation began, plates were checked approximately every 5 h (morning, midday, and evening). Continual monitoring occurred from pupation to emergence of adults in the same ~5 h increments for measuring total time it took for flies to reach the adult stage. This was performed in six replicates per group (strain and galbut virus-infection status). Data were not normally distributed (Shapiro–Wilk test); thus, a Wilcoxon test was performed to determine statistical significance between groups.

*Pseudomonas aeruginosa* oral challenge. Flies were challenged orally with *Pseudomonas aeruginosa*, as adapted from Lutter et al. [41]. An overnight culture of *P. aeruginosa* (strain PAO1) was grown in a 200 mL culture Brain Heart infusion (BHI) broth incubated at 220 rpm at 37 °C. The following day, the culture was centrifuged at 4200× *g* for 5 min until a loose pellet was formed. Excess supernatant was decanted, and culture was resuspended to an OD_600nm_ of ~7 using a sterile 5% sucrose solution. Autoclaved filter disks were inoculated with 290 µL of the *P. aeruginosa* solution. Disks were placed on 5% sucrose agar vials. Control disks were inoculated with the 5% sucrose solution. Twelve flies that had been starved for 5 h were placed in the bacteria-containing vials for each replicate. Flies that died by the end of the first day were censored from further analysis since their deaths were likely due to starvation stress. Survival of flies was monitored daily for 12 days. Statistical analysis was performed using the R survival package [38]. A total of three technical replicates was performed.

Intrathoracic microbial pathogen challenges. The following pathogen challenges were performed through intrathoracic microinjection. All experimental injections were performed in three biological replicates (12 flies per replicate) per technical replicate, and a total of two technical replicates were performed for each pathogen. An exception is the *Staphylococcus aureus* challenge, which was performed in three technical replicates. Control injections with 1× phosphate-buffered saline (PBS) were performed in parallel. Flies were checked at 10–12 h post injection, and any flies that were dead at this point were assumed to have died from injection. Additionally, any flies that died from non-natural causes (for example, after getting stuck in the media) were also censored from analysis. Injected volumes, inoculum dose, and subsequent intervals for checking fly survival are stated below for the respective pathogen.

*Pseudomonas aeruginosa*: Flies were microinjected with *P. aeruginosa* (strain PAO1). A culture was started by inoculating 150 mL of BHI broth and incubated at 220 rpm overnight at 37 °C. The following day, the culture was centrifuged at 4200× *g* for 5 min until a loose pellet was formed. Excess supernatant was decanted, and the culture was resuspended to an OD_600nm_ of 0.03 using 1× PBS. Flies were injected with 9.2 nL of this diluted *P. aeruginosa* culture, which corresponds to ~100 colony forming units (CFU) [42]. Flies were incubated overnight and checked at 24 h post injection, 28 h post injection, and every 2 h from 28 to 42 h post injection. After 42 h post injection, flies were checked at one final time point of 52 h post injection, at which any living flies were censored from downstream statistical analyses.

*Staphylococcus aureus*: Flies were microinjected with *S. aureus* (strain XEN36, Perkin Elmer). A culture was obtained by inoculating 150 mL BHI broth and stirred at 220 rpm overnight at 37 °C. The following day, the culture was centrifuged at 4200 g for 5 min until a loose pellet was formed. Excess supernatant was decanted, and the culture was resuspended to an OD_600nm_ of 0.1 using 1× PBS. Flies were injected with 23 nL of this diluted *S. aureus* culture, which corresponds to ~100 CFU [43]. Flies were checked daily until 8 days post injection, at which point any living flies were censored from downstream statistical analyses.

Drosophila C virus: Drosophila C virus (DCV) stocks were provided by the Andino lab at the University of California San Francisco. DCV stocks were amplified and titrated on *Drosophila* S2 cells. DCV infections of flies were performed as previously described [44]. Flies were microinjected with DCV at a titer of 100 × 50% tissue culture infective dose units (TCID_50_) in a total volume of 50 nL. Flies were checked daily until 14 days post injection, at which point any living flies were censored from downstream analyses.

*Candida albicans*: *Candida albicans* challenge was performed as previously described [45]. *C. albicans* (strain SC5314) was obtained from ATCC. A yeast extract peptone dextrose (YPD) agar plate was streaked from the frozen glycerol stock and incubated at 30 °C for 18 h. Then, 150 mL of YPD broth was inoculated with a single colony from the YPD plate and incubated at 220 rpm overnight at 30 °C until the culture was at an OD_600 nm_ of ~1. The culture was centrifuged at 4200× *g* for 5 min until a loose pellet was formed, which was resuspended using 1x PBS. Yeast cells were counted with a cytometer and diluted to 107 cells/mL. Flies were microinjected with 50 nL (~500 cells) of this dilution. Flies were incubated at 30 °C and were checked daily until 6 days post injection, at which point any living flies were censored from analyses.

Multiple hypothesis correction. We adjusted the *p*-value significance threshold to account for multiple hypothesis testing. This study tested the hypotheses that galbut virus infection influences 12 traits: microbiome composition, lifespan, numbers of adult offspring, offspring sex ratio, numbers of eggs laid, times to pupation and eclosion, and survival following challenge by four pathogens (with *Pseudomonas aeruginosa* challenge via two routes). These traits were each measured in two strains and two sexes, so we tested 48 hypotheses in total. The Bonferroni adjusted significance threshold was therefore 0.05/48 = 1.0 × 10^−3^. 

## 3. Results

### 3.1. Confirmation of Galbut virus-Infection Status and Galbut Virus RNA Levels in Individual Flies

**Figure 1:**
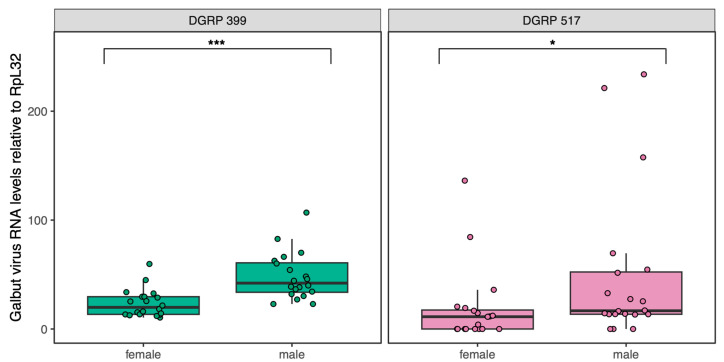
Relative galbut virus RNA levels in flies from two DGRP strains. Boxplots depicting galbut virus RNA levels relative to levels of RpL32 mRNA (2^−ΔCt^ method) in DGRP 399 (green) and DGRP 517 (pink) adult flies (*n* = 10 per strain and sex). * *p* < 0.05; *** *p* < 0.001. Statistical tests described in Materials and Methods.

We measured fitness traits in flies from DGRP lines 399 and 517, as we had previously established populations of these lines that were persistently infected by galbut virus [4]. There was no indication that these lines were resistant to infection, but we nevertheless first verified virus persistence in the populations. We quantified galbut virus RNA levels using qRT-PCR in twenty 3–5-day-old flies from each line (10 male and 10 female) and normalized levels to those of ribosomal protein L32 messenger RNA (RpL32 mRNA; Figure 1). Galbut virus RNA levels were higher than those of highly expressed RpL32 mRNA in all cases (Figure 1). Median galbut virus RNA levels were 2.3-fold higher in DGRP 399 flies than in DGRP 517 flies (*p* = 1.6 × 10^−2^) although a few DGRP 517 flies had the highest galbut virus RNA levels (Figure 1). Galbut virus RNA levels were 2.1-fold higher in DGRP 399 males than in females (*p* = 4.2 × 10^−5^) and 1.5-fold higher in DGRP 517 males than in females (*p* = 1.3 × 10^−2^). Therefore, these populations remained persistently infected at 100% prevalence, and galbut virus RNA levels varied as a function of DGRP strain and sex, with levels generally higher in males and in DGRP 399 flies. Flies from the parental DGRP 399 and 517 lines were consistently negative for galbut virus by qRT-PCR.

### 3.2. Galbut Virus Infection Does Not Have Significant Impacts on Predominant Microbiome Constituents

The microbiome composition of *D. melanogaster* can alter fitness [37]. It is also possible that viral and bacterial constituents of the microbiota can interact [46]. Commensal bacteria can also vary by DGRP background when reared under the same conditions [36]. We therefore tested whether the microbiomes of these populations varied as a function of galbut virus-infection status. Our goals were to assess whether microbiome differences could underlie differential phenotypes in flies with and without galbut virus and whether galbut virus infection was altering microbiome composition. 

**Figure 2:**
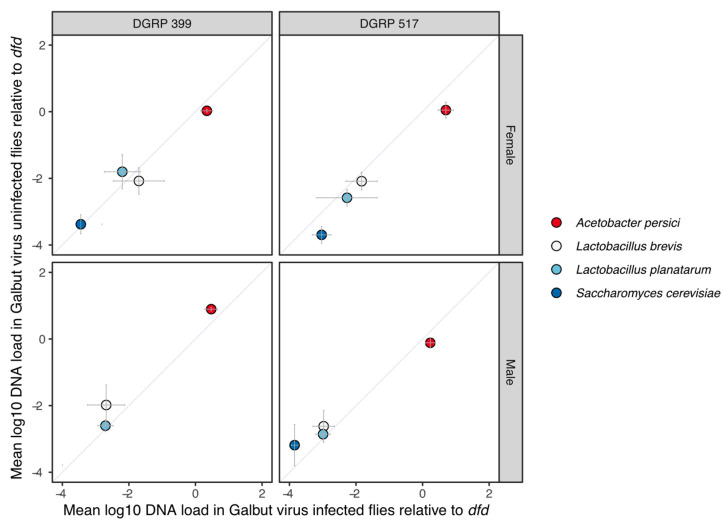
Galbut virus infection did not alter levels of major microbiome constituents in flies. Relative amounts of DNA from predominant microbiome constituents in galbut virus-infected and uninfected flies were measured via qPCR from three replicate pools of 10 flies per pool per strain per sex. Mean DNA loads relative to single copy *dfd* gene are plotted, and crossbars indicate standard deviations of replicates. No statistically significant differences between galbut virus-infected and uninfected flies were identified using a Wilcoxon test.

Exploratory 16S ribosomal RNA gene sequencing of DGRP 399 and 517 flies reared in our lab revealed that these lines had similar microbiome compositions, the major constituents of which included *Acetobacter persici*, *Lactobacillus (L.) brevis*, and *L. planatarum*. We quantified DNA levels of these bacteria and of *Saccharomyces cerevisiae*, which is fed to the flies, by qPCR using previously designed primers. DNA copy numbers were normalized to the single copy host gene *deformed* (*dfd*), as previously described [36]. The relative abundances of the different microbes were similar in DGRP 399 and 517 flies and in males and females (Figure 2). Galbut virus infection did not produce any statistically significant differences in DNA levels of these taxa in any of the groups (Figure 2). This indicated that galbut virus infection did not measurably change microbiome composition and that any fitness effects of galbut virus infection were likely not mediated by changes in microbiome composition.

### 3.3. Galbut Virus Minimally Impacts Drosophila Lifespan and Fecundity

**Figure 3:**
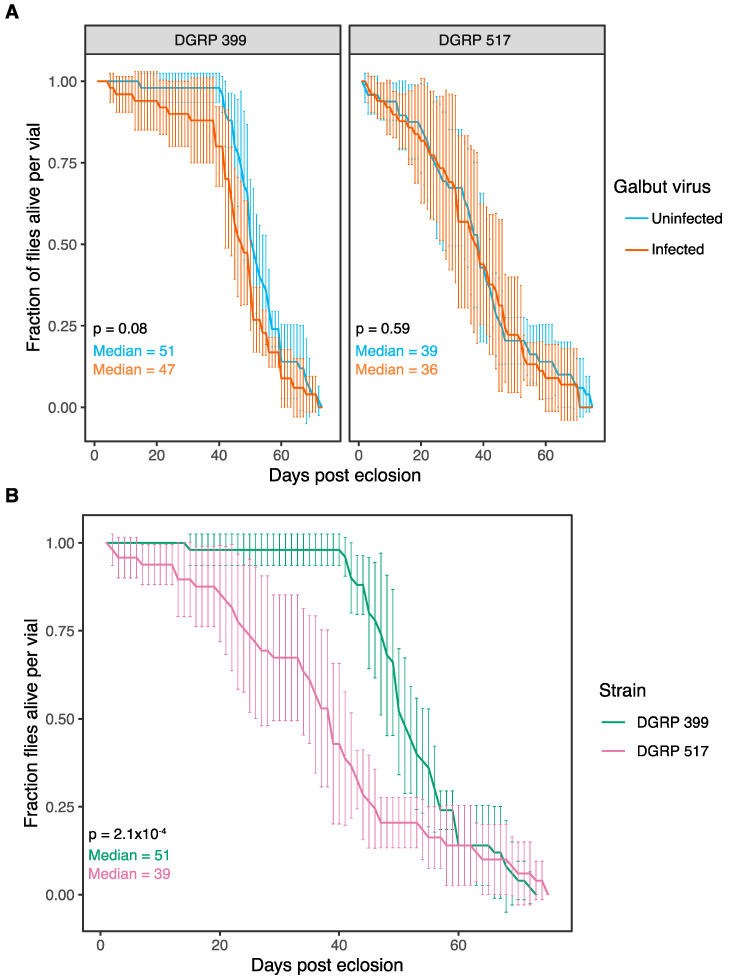
Galbut virus-infected flies do not exhibit changes in lifespan. (**A**) Survival of DGRP 399 and 517 flies with or without persistent galbut virus infection. The mean and standard deviation of biological replicates is plotted. (**B**) Data as in A but plotted to facilitate comparison of DGRP strains. Statistical tests described in Materials and Methods.

We compared the lifespan, fecundity, and developmental speed of galbut virus-infected and uninfected flies [37,47]. Vials of newly eclosed adults (*n* = 5 replicate vials per experimental group) were housed together in groups of 10 flies (5 males, 5 females). The median lifespan of galbut virus-infected flies was not significantly different than that of uninfected flies (Figure 3A). In contrast, the lifespan of flies from the two DGRP strains were different: the median lifespan of DGRP 517 flies was 12 days shorter than that of DGRP 399 flies (*p* = 2.1 × 10^−4^, Figure 3B). 

**Figure 4:**
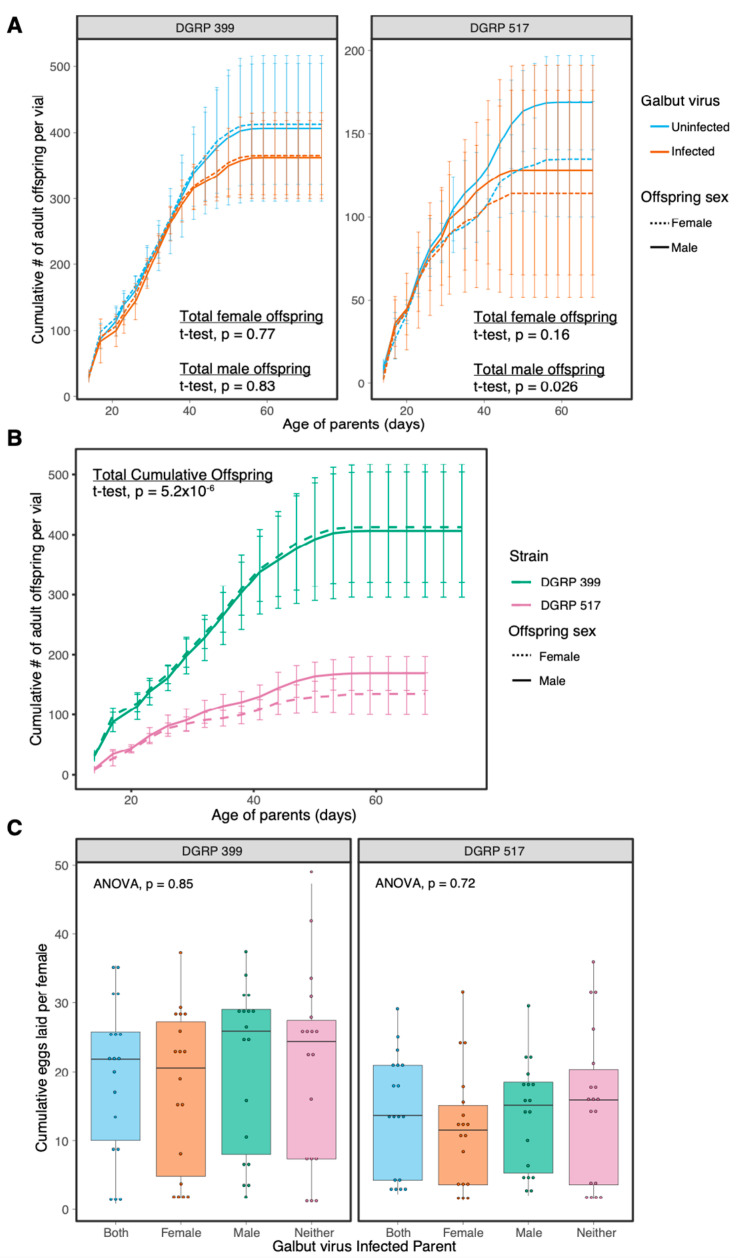
Impact of galbut virus infection on fecundity. (**A**) Galbut virus-infected and uninfected flies were housed in batches of five males and five females per vial, and cumulative number of female and male offspring per vial were counted. The mean and standard deviation of biological replicates are plotted. (**B**) Data as in panel A but plotted for comparison of DGRP strains (data from galbut virus-uninfected flies shown). (**C**) Ten male and ten female flies 3–5 days post eclosion were crossed with different combinations of galbut virus-infected mothers or fathers. The cumulative number of eggs laid per female over three days is depicted for individual replicates as points and summarized with boxplots. Statistical tests described in Materials and Methods.

We compared fecundity of infected and uninfected flies by counting total adult offspring in vials containing five male and five female parents. Galbut virus-infected flies produced the same number of cumulative offspring as their uninfected counterparts (*t*-test; DGRP-399: female offspring *p* = 0.77, male offspring *p* = 0.83; DGRP-517: female offspring: 0.16, male offspring: *p* = 0.026 Figure 4A). Galbut virus infection did not significantly change offspring sex ratios (*t*-test; DGRP 399: *p* = 0.63, DGRP 517: 0.75, Figure S1). As with average lifespan, the two DGRP strains exhibited differences in total offspring produced: DGRP 399 females produced on average 2.7× more offspring than DGRP 517 females (*p* = 5.2 × 10^−6^, Figure 4B).

We recorded the cumulative number of eggs laid over three days when one or both parents were infected by galbut virus. There were no significant differences in the number of eggs laid when either or both parents were infected with galbut virus (ANOVA, DGRP 399: *p* = 0.85, DGRP 517: *p* = 0.72; Figure 4C). 

**Figure 5:**
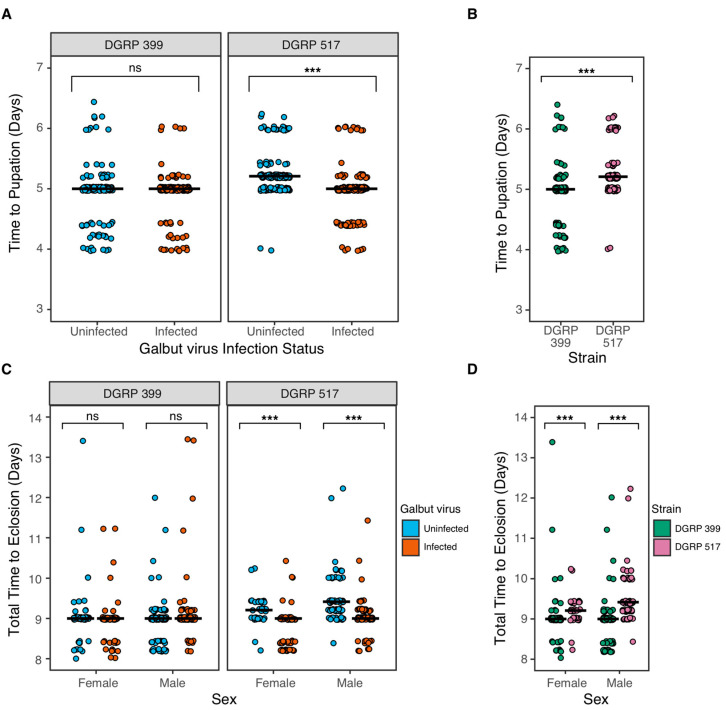
Galbut virus-infected DGRP 517 flies developed faster than their uninfected counterparts. (**A**) The time to pupation of individual DGRP 399 or DGRP 517 flies is plotted, and the median time is indicated by a crossbar. (**B**) Data as in A but plotted to enable comparison between DGRP strains. (**C**) The time between oviposition and eclosion for individual flies is indicated. (**D**) Data as in C but plotted to enable comparison between DGRP strains. ns, not significant; *** *p* < 0.001. Statistical tests described in Materials and Methods.

We compared the developmental speed of galbut virus-infected and uninfected flies by collecting eggs and monitoring the times from oviposition to pupation and oviposition to adulthood (Figure 5). DGRP 399 flies pupated in ~5 days and eclosed in ~9 days regardless of galbut virus-infection status (Figure 5A,C). DGRP 517 flies infected with galbut virus pupated on average 7 h faster than uninfected flies (Wilcoxon, *p* = 2.2 × 10^−16^; Figure 5A). DGRP 517 infected females and males eclosed on average 10 and 12 h faster than their uninfected counterparts (Wilcoxon, female *p* = 5.6 × 10^−8^, male *p* = 7.0 × 10−^14^; Figure 5C). As was the case with other phenotypes, development speed also varied as a function of DGRP background, with DGRP 399 flies pupating on average 7 h faster than DGRP 517 flies (Figure 5B) and eclosing on average 13 h faster (Figure 5D). 

### 3.4. Galbut Virus Alters the Susceptibility of Flies to Viral, Bacterial, and Fungal Pathogens

The microbiota present in a host can influence the outcome of subsequent infections [48,49,50,51]. Moth-infecting partitiviruses changed their host’s ability to withstand infection by a pathogenic nucleopolyhedrovirus [28]. We hypothesized that galbut virus infection might alter the ability of flies to resist or tolerate infection by pathogenic microbes, which could alter the fitness of galbut virus-infected flies. To test this hypothesis, we challenged galbut virus-infected and uninfected flies with viral, bacterial, and fungal pathogens.

**Figure 6:**
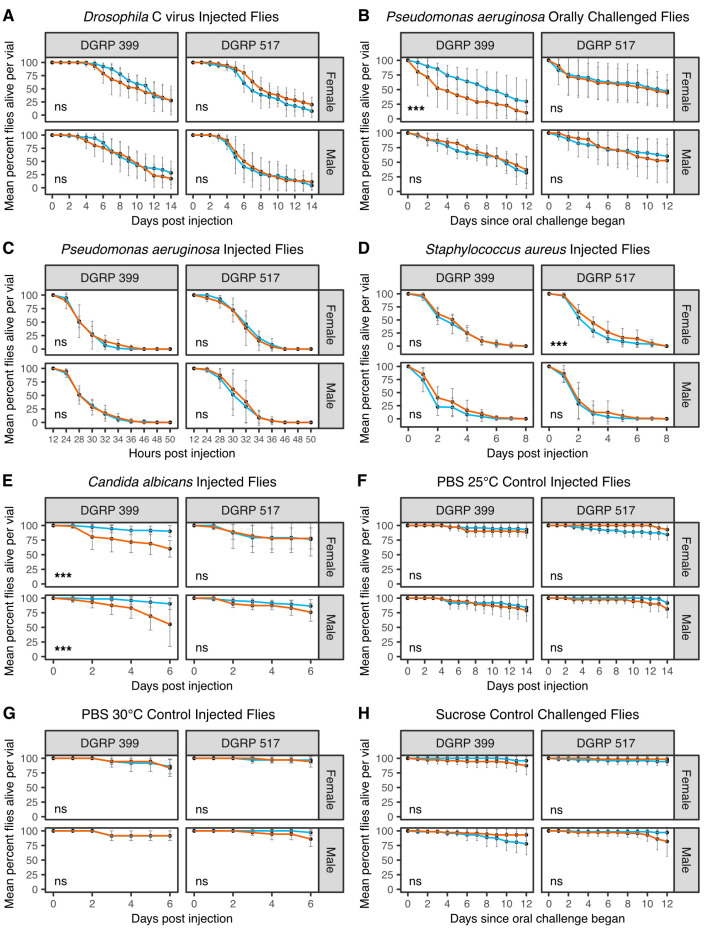
Galbut virus alters pathogen susceptibility of some flies. Survival of galbut virus-infected and uninfected flies following (**A**) intrathoracic injection with 100 TCID_50_ units of Drosophila C virus (DCV), (**B**) ingestion of *Pseudomonas aeruginosa*, (**C**) injection of ~100 CFU of *P. aeruginosa*, (**D**) injection with ~100 CFU of *Staphylococcus aureus*, and (**E**) injection with ~500 *Candida albicans* cells. (**F**–**H**) Survival of flies following control inoculations. Flies were either microinjected with phosphate-buffered saline (PBS) and stored at 25 °C (**F**) or 30 °C (**G**) or ingested sucrose (H). Galbut virus-infected flies are depicted in orange, and uninfected flies are in blue. ns, not significant; *** *p* < 0.001. Statistical tests described in Materials and Methods.

We first tested whether pre-existing galbut virus infection altered fly survival following infection by Drosophila C virus (DCV) [52]. Flies were challenged with 100 TCID_50_ units of DCV through intrathoracic microinjection and checked daily for survival. There was no significant difference in the survival of galbut virus-infected and uninfected flies (Figure 6A). These DGRP strains are both *Wolbachia* -negative, so improved survival could not be attributed to the known protective effects of *Wolbachia* against DCV [53,54,55,56].

We next challenged flies orally with *Pseudomonas aeruginosa*. Galbut virus-infected DGRP 399 female flies were more susceptible to *P. aeruginosa* bacterial challenge (Figure 6B; *p* = 4.5 × 10^−6^). Although ingestion is a more natural route of infection than microinjection, there is less experimental control over the ingested dose, which can decrease reproducibility [42]. We therefore also injected flies with ~100 CFU of *P. aeruginosa*. Flies injected with *P. aeruginosa* died faster than those that ingested the pathogen, with most flies dead by 36 h post injection (Figure 6C). Galbut virus-infected DGRP 399 females no longer died faster than their uninfected counterparts when microinjected with *P. aeruginosa* (Figure 6C; *p* = 0.14). This suggests that interactions between galbut virus and *P. aeruginosa* may depend on the route of infection.

Since the *Drosophila* innate immune system responds differently to Gram-negative and Gram-positive bacteria [57], we continued our pathogen challenges by microinjecting flies with *Staphylococcus aureus*. When flies were microinjected with ~100 CFU *S. aureus*, galbut virus-infected DGRP 517 females survived slightly longer than their galbut virus-uninfected counterparts (*p* = 6.8 × 10^−4^; Figure 6D).

As a final pathogen challenge, we injected flies with ~500 cells of the fungal pathogen *Candida albicans* [45]. Both male and female DGRP 399 galbut virus-infected flies died faster than their uninfected counterparts following *C. albicans* challenge (Figure 6E; DGRP-319 females *p* = 6.5 × 10^−6^ and DGRP-319 males *p* = 3.5 × 10^−5^). No significant differences were observed for DGRP 517 flies (Figure 6E).

Control injections were performed with PBS to confirm fly death was not due to injection trauma. Injected flies were housed alongside experimental injections at either 25 °C (Figure 6F) or 30 °C (Figure 6G). No statistical differences were noted in fly survival, and in general, fly mortality was low in these samples. Likewise, a control for oral inoculation was performed where flies were fed sucrose in place of *P. aeruginosa* (Figure 6H). Minimal death was observed, and there were no significant differences in survival between groups.

## 4. Discussion

A major goal of this study was to understand why galbut virus, despite a high rate of vertical transmission (~100% from both parents), is maintained at a worldwide prevalence of only ~60%. We hypothesized that galbut virus infection might inflict enough of a net negative fitness cost that resistant flies would experience a survival benefit. To test this hypothesis, we quantified multiple components of fitness in two genetic backgrounds. 

Galbut virus infection did not significantly decrease lifespan nor fecundity of infected flies even during experiments that lasted much longer than the natural lifespan of *D. melanogaster*, which is estimated to be a week or less in the wild [58] (Figure 3 and Figure 4). There was also no impact on the number of eggs laid by young females over three days (Figure 4). Examples of partitiviruses altering the reproductive output of their hosts include a partitivirus enhancing fecundity in *Cryptosporidium* [59], a reduction of spores from a partitivirus-infected fungus [60], and partitiviruses infecting *Spodoptera* moths that decreased hatchling numbers [60]. 

DGRP 517 flies infected by galbut virus pupated and reached adulthood faster than uninfected flies (Figure 5). An initial assumption would be that a faster developmental time, in combination with the short life of flies in the wild [58], would confer a fitness benefit. However, flies selected for faster development exhibited fitness trade-offs such as reduced body weight and size, decreased resistance to starvation and desiccation, and an overall lower egg output [61]. This highlights the difficulty of extrapolating total fitness from singly measured traits [15]. 

For the most part, galbut virus-infected and uninfected flies survived similarly following infection by microbial pathogens (Figure 6). Galbut virus-infected DGRP 399 females exhibited decreased survival following ingestion but not injection of *Pseudomonas aeruginosa*. This difference may not be surprising, as the gut epithelial immune response has key differences compared to responses to systemic infection [62]. DGRP 399 flies of both sexes exhibited increased sensitivity to the fungal pathogen *Candida albicans*. In *Drosophila*, the common microbiome constituent *Lactobacillus planatarum* decreased mortality of a fungal pathogen (*Diaporthe* sp.) by mitigating fungal toxicity and altered fly behavior to reduce infection risk [63]. No significant changes in the DNA levels of *L. planatarum* or other major microbiome constituents were observed in galbut virus-infected flies (Figure 2). 

It is difficult to assess the net impact of these separately measured traits with possibly offsetting impacts on fitness. In some cases, galbut virus-infected flies would be predicted to have decreased fitness, for instance, due to decreased survival following fungal infection (Figure 6E). In other cases, trait differences such as faster development might increase the relative fitness of galbut virus-infected flies (Figure 5). For most measured traits, differences associated with galbut virus infection were smaller than those attributable to different DGRP strain and sex. Nevertheless, selection can act on small differences in relative fitness, and it is possible that in aggregate, galbut virus infection reduces fitness. Galbut virus is highly prevalent, exhibits a broad tissue distribution, and exists as a lifelong infection, so small phenotypic changes should not necessarily be interpreted as insignificant ones [64]. Additional laboratory and field-based studies that track galbut virus–*Drosophila* dynamics will shed further light on the extent to which this virus and similar persistent viruses shape the evolution of their hosts in cryptic but possibly important ways.

## Data Availability

Computational scripts and underlying data for analysis of experiments can be found at https://github.com/scross92/galbut_fitness_analysis (accessed on 31 January 2023).

## Supplementary Materials

The following supporting information can be downloaded at: https://www.mdpi.com/article/10.3390/v15020539/s1, Figure S1: Galbut virus infection does not influence adult offspring sex ratio; Table S1: Primers used for quantifying levels of galbut virus and microbiome constituents.
