# Supplementary material for: Galbut Virus Infection Minimally Influences Drosophila melanogaster Fitness Traits in a Strain and Sex-Dependent Manner
Source: Viruses. 2023 Feb 15;15(2):539. doi: 10.3390/v15020539 (PMC9965562; doi:10.3390/v15020539)
Supplement: Supplementary file 1 [file viruses-15-00539-s001.zip › viruses-2133245-supplementary.pdf]

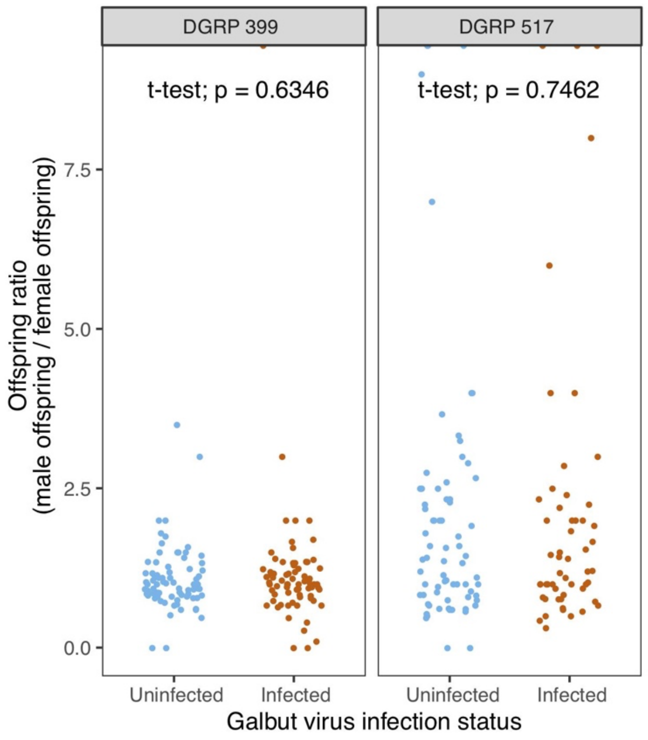

Supplemental Figure S1. Galbut virus infection does not influence adult offspring sex ratio. Offspring collected from groups of galbut virus infected or uninfected parents from DGRP 399 and 517 strains every 14 days. Offspring sex ratios from each time point were calculated by dividing total male offspring by total female offspring. No statistical significance was measured in either strain.

Supplemental Table S1: Primers used for quantifying levels of galbut virus and microbiome constituents.

**Target**	**Forward (5′-3′)**	**Reverse (5′-3′)**	**Reference**
Galbut virus	CCGTGAAGCAAGGAATCAAT	TGCCGATTTTCTGCTCTTTT	Cross et al: https://doi.org/10.1128/JVI.01070-20
RpL32 mRNA	TGCTAAGCTGTCGCACAAATGG	TGCGCTTGTTCGATCCGTAAC	Cross et al: https://doi.org/10.1128/JVI.01070-20
Acetobacter spp.	TAGCTAACGCGATAAGCACA	ACAGCCTACCCATACAAGCC	AM Early et al: https://doi.org/10.1371/journal.pone.0170332
Lactobacillus brevis	TCAGTTTTGAGGGGCTTACCTCTCT	GGCATCCACCATGCGCCCTT	AM Early et al: https://doi.org/10.1371/journal.pone.0170332
Lactobacillus planatarum	TGCGGCTGGATCACCTCCTTTC	ACTGGTTCGGTTCCAATGGGCC	AM Early et al: https://doi.org/10.1371/journal.pone.0170332
Saccharomyces cerevisiae	AGGAGTGCGGTTCTTTG	TACTTACCGAGGCAAGCTACA	H Chang et al: https://doi.org/10.1016/j.mimet.2007.08.013
deformed (*dfd*)	GTAGCGAAGAAACCCACCAA	ACGCTCCACTCACCTCATTC	AM Early et al: https://doi.org/10.1371/journal.pone.0170332
